# Supplementary material for: Profiling of insulin-resistant kidney models and human biopsies reveals common and cell-type-specific mechanisms underpinning Diabetic Kidney Disease
Source: Nat Commun. 2024 Nov 19;15:10018. doi: 10.1038/s41467-024-54089-1 (PMC11576882; doi:10.1038/s41467-024-54089-1)

Supplementary Figures for

Profiling of insulin-resistant kidney models and human biopsies reveals common and cell-type-specific mechanisms underpinning Diabetic Kidney Disease

Abigail C Lay, Van Du T Tran, Viji Nair, Virginie Betin, Jennifer A Hurcombe, Alexandra F Barrington, Robert JP Pope, Frédéric Burdet, Florence Mehl, Dmytro Kryvokhyzha, Abrar Ahmad, Matthew C Sinton, Philip Lewis, Marieangela C Wilson, Rajasree Menon, Edgar Otto, Kate J Heesom, Mark Ibberson, Helen C Looker, Robert G Nelson, Wenjun Ju, Matthias Kretzler, Simon C Satchell, Maria F Gomez, Richard JM Coward, BEAt-DKD consortium

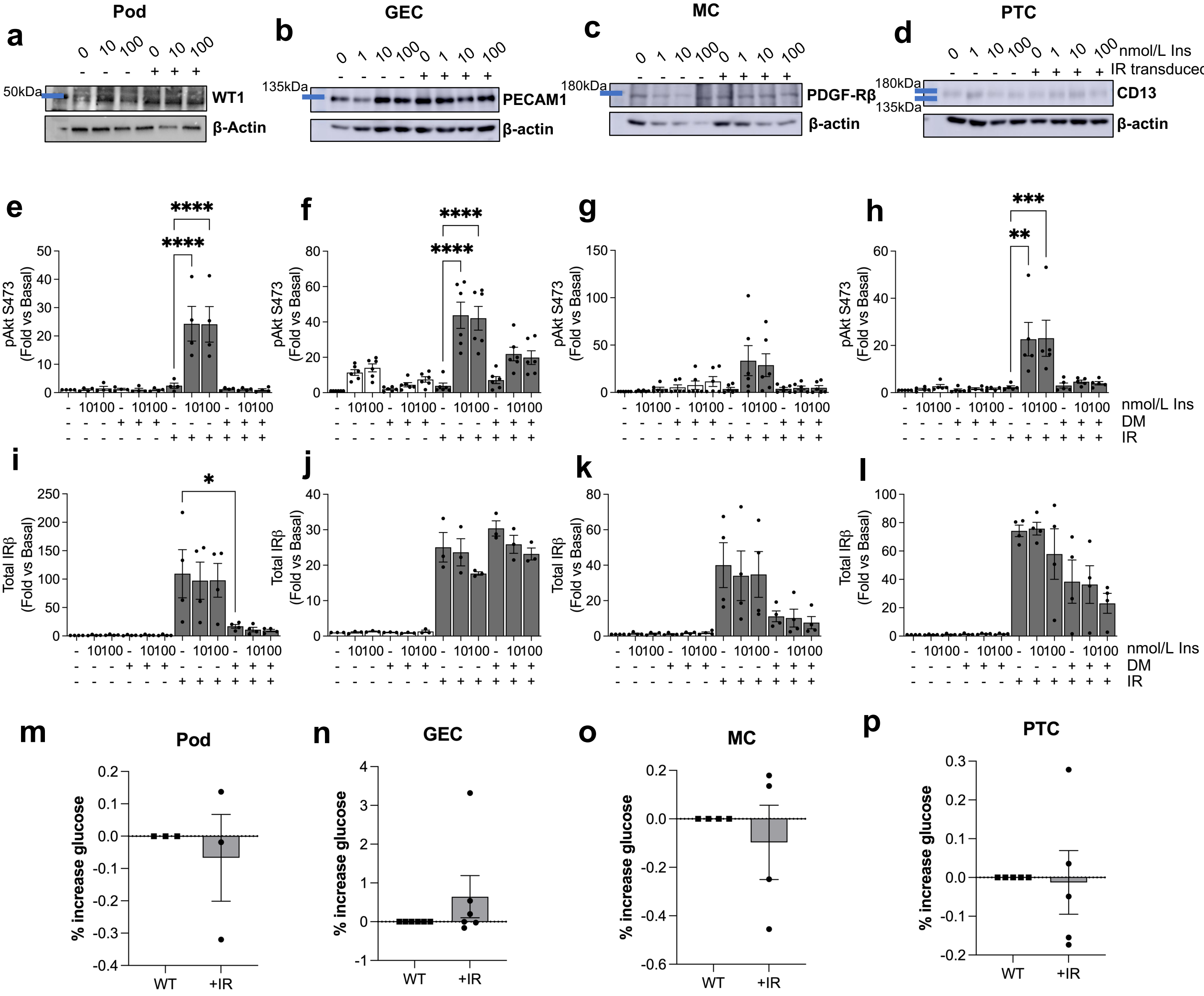

Supplementary Fig. 1: Overview of cell models

Conditionally immortalised human **a** podocytes (Pods), **b** glomerular endothelial cells (GECs), **c** mesangial cells (MCs) and **d** proximal tubular cells (PTCs) were studied *in vitro* in a basal and insulin resistant environment (consisting of 1ng/ml TNFα, 1ng/ml IL-6, 25mM glucose and 100nmol/L insulin, 'DM'). Insulin-sensitive cell lines were established via stable overexpression of the human insulin receptor (IR). Where indicated, cells were further stimulated with insulin for 15 minutes at 10 or 100nmol/L. **a-d** Western blotting to show expression of marker proteins. Densitometry values for Akt phosphorylation (S473) in **e** Pods (*n*=4), **f** GECs (*n*=6), **g** MCs (*n*=6) and **h** PTCs (*n*=5) and Total IRβ in **i** Pods (*n*=4), **j** GECs (*n*=4), **k** MCs (*n*=4) and **l** PTCs (*n*=4) under the indicated conditions, **p* < 0.05, ***p* < 0.01, ****p* < 0.001, *****p* < 0.0001, one-way ANOVA with Tukey's multiple comparison test. Percentage change in cellular uptake of [3H]2-deoxy-D-glucose in IR-transduced ('+IR') vs wild—type ('WT') **m** Pods (*n*=3), **n** GECs (*n*=6), **o** MCs (*n*=4) and **p** PTCs (*n*=5), ns, two-tailed *t*-test.

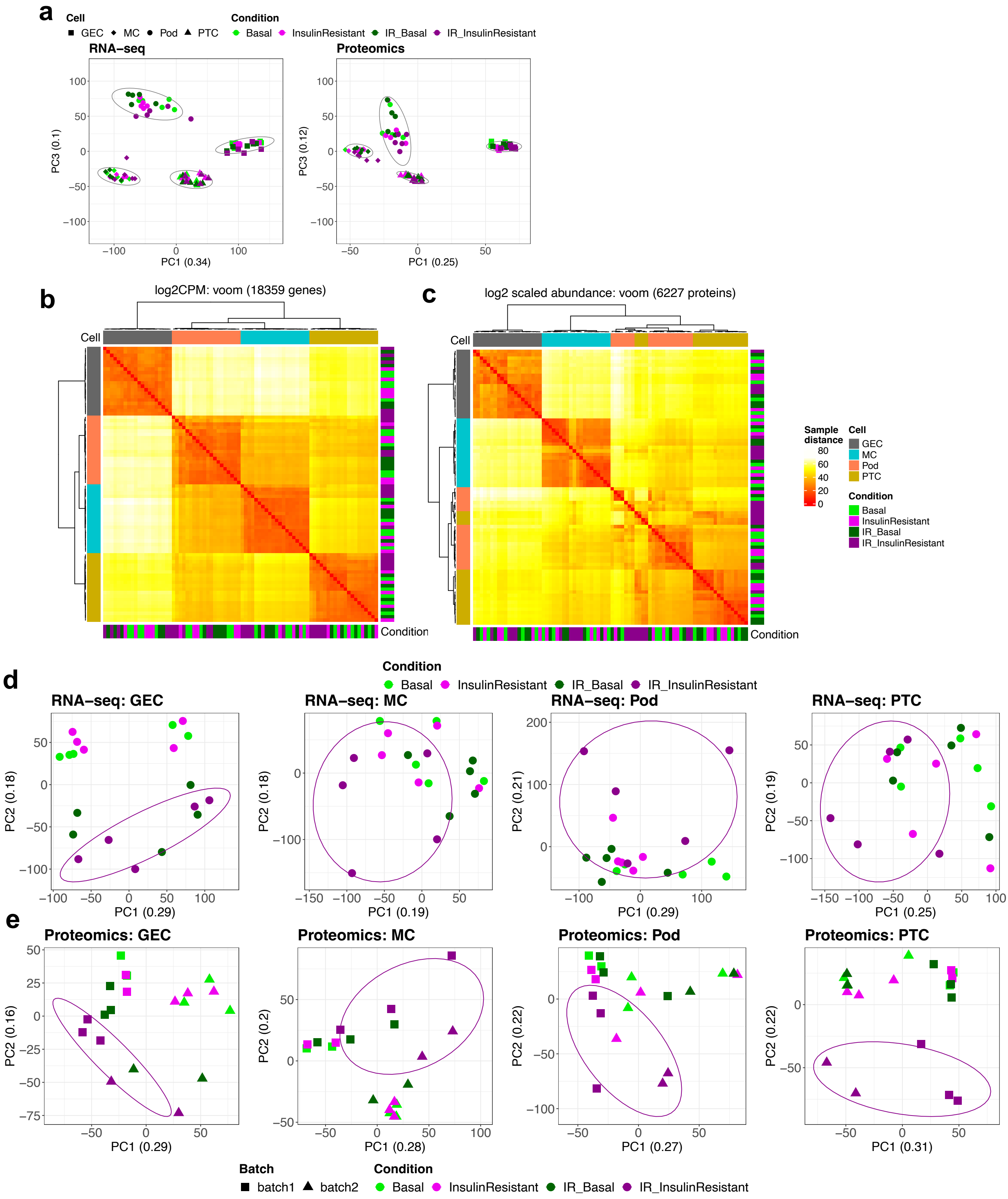

Supplementary Fig. 2: Overview of transcriptomics and proteomics data from insulin-sensitive and insulin-resistant human cell lines

RNA and protein were simultaneously isolated from podocytes (Pods), Glomerular Endothelial Cells (GECs), Mesangial Cells (MCs) and Proximal Tubular Cells (PTCs) under basal and insulin-resistant conditions, with and without additional IR-transfection (n=5/condition). **a** Principal component analysis (PCA) of >18 000 transcripts identified by RNAseq and >6 000 proteins, detected across all cell lines and conditions studied, demonstrating the primary clustering of samples by cell type. Sample-to-sample Euclidian distance heatmaps for **b** transcriptomics and **c** proteomics data. PCA of **d** transcriptomic and **e** proteomics data, performed on individual cell types, demonstrates separation of highly insulin-resistant samples.

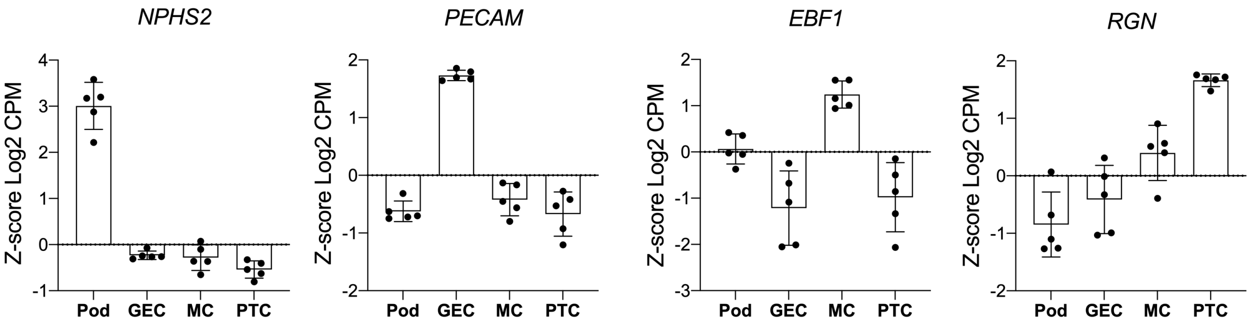

Supplementary Fig. 3: Examples of cell-type-specific genes detected in transcriptomics data from human cell lines

Expression of *NPHS2*, *PECAM*, *EBF1* and *RGN* in conditionally-immortalised human Podocytes (Pods), Glomerular endothelial cells (GECs), Mesangial Cells (MCs), and proximal tubular cells (PTs).

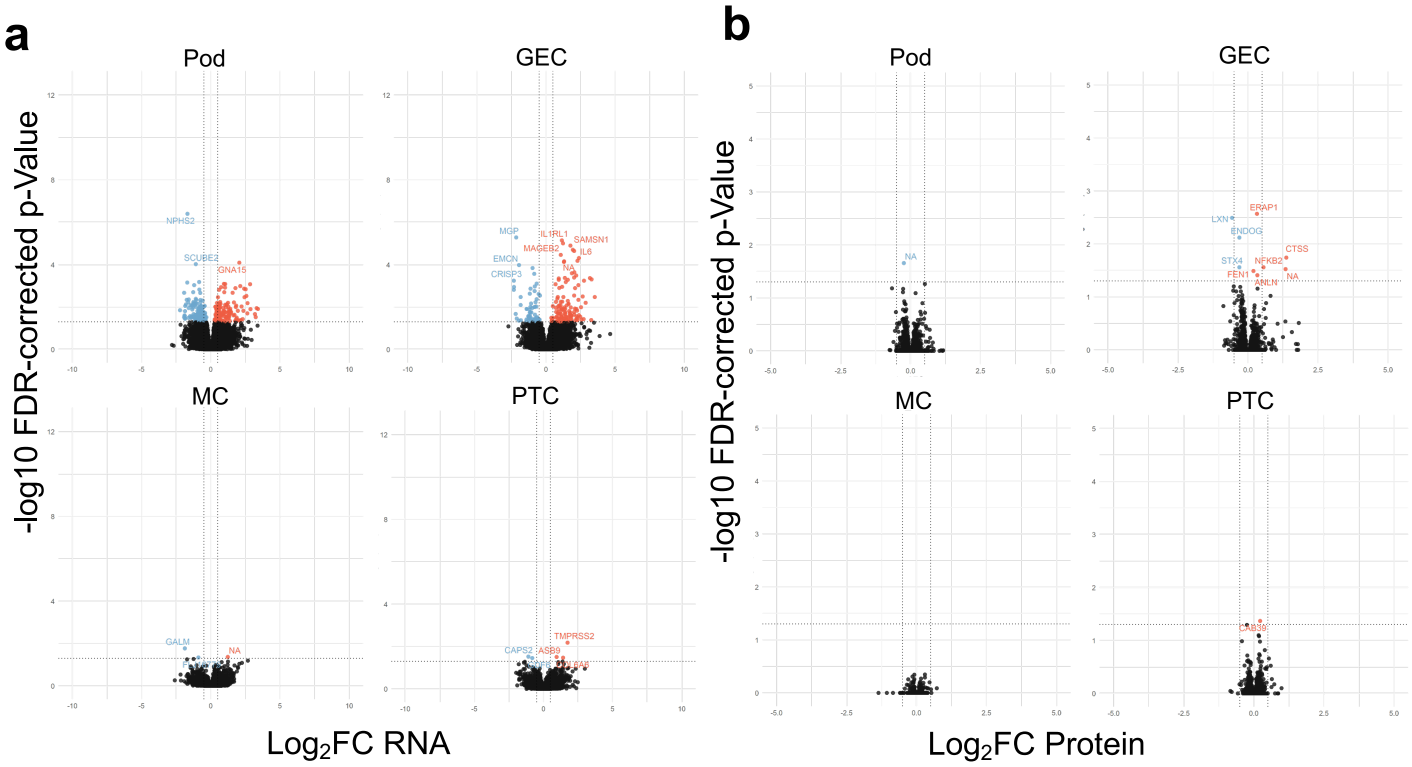

Supplementary Fig. 4: Transcriptome and proteome changes in non-insulin receptor transfected basal v insulin-resistant kidney cell lines

a Transcripts and **b** Proteins differentially expressed podocyte (Pod), glomerular endothelial cell (GEC), Mesangial cell (MC) and Proximal tubular Cell (PTC) lines in diabetic (insulin-resistant) environment vs basal conditions, with examples of significantly regulated molecules highlighted (FDR<0.05).

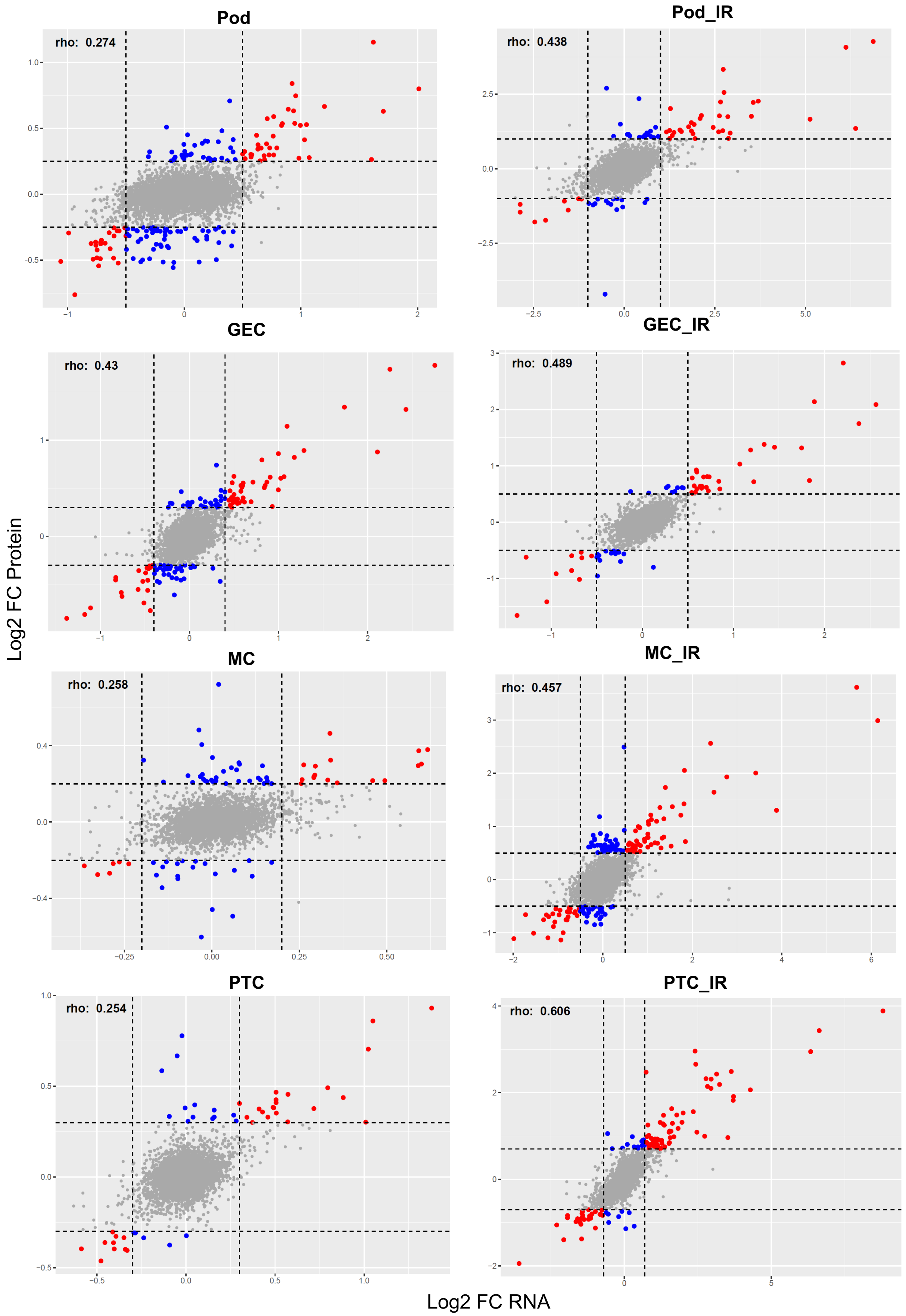

Supplementary Fig. 5: Comparison between regulation at the transcript and protein level within in insulin-resistant vs insulin-sensitive cell lines

Correlation plots demonstrating regulation at transcript- and protein- level indicating stronger correlation between regulation at the transcript and protein level in response to a diabetic environment in our highly-insulin sensitive cell model (i.e., IR-transfected cell lines)

Cell type	R^2X	R^2Y	Q^2	t-test (n=1000)
**Pod**	0.394	0.971	0.753	$p < 0.001$, mean = -0.111, SD = 0.411
**GEC**	0.368	0.958	0.416	$p < 0.001$, mean = -0.196, SD = 0.612
**MC**	0.398	0.977	0.621	$p < 0.001$, mean = -0.117, SD = 0.426
**PTC**	0.496	0.982	0.841	$p < 0.001$, mean = -0.195, SD = 0.580

Supplementary Fig. 6: Overview of Consensus Orthogonal Partial Least Squares Discriminant Analysis (OPLS-DA) model

Consensus OPLS modelling was performed on proteomics and RNAseq data tables, which were all autoscaled prior to the analysis, for each cell type independently. The models were computed with 2 latent variables, 1 predictive and 1 orthogonal. The quality of the model was assessed by R^2 and Q^2 values, which define the portion of data variance explained by the model and the predictive ability of the model, respectively. The Q^2 value was computed by a K-fold cross validation (K=7). To ensure the validity of the model, a series of 1,000 permutation tests were carried out by mixing randomly the original Y response (basal vs insulin resistant). A t-test was performed to ensure that the true model Q^2 value was clearly distinguished and statistically different from the random model distribution. The variable relevance to discriminate between the two conditions was evaluated using the variable importance in projection (VIP) parameter, which reflects the importance of variables both with respect to the response and to the projection quality.

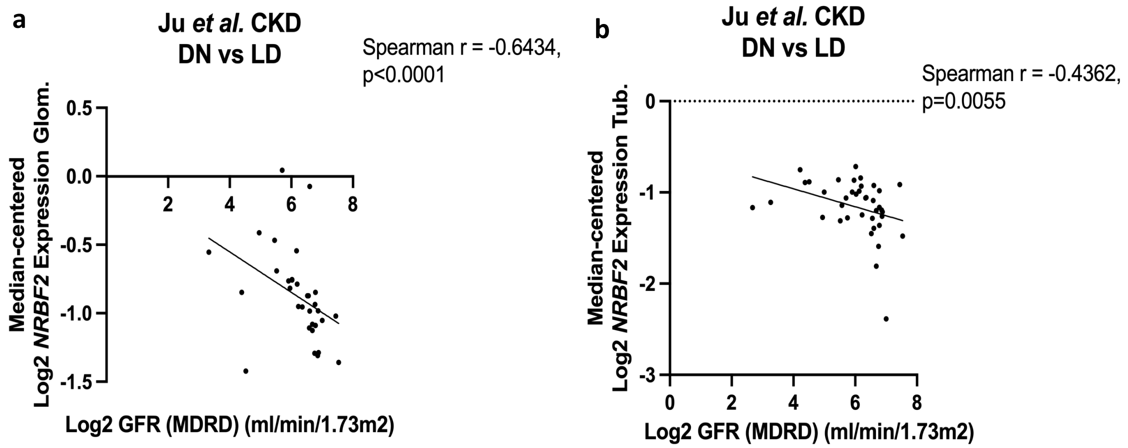

Supplementary Fig. 7: *NRBF2* expression in human late-stage DKD

Human microarray data (Log2) and eGFR from the 'Ju-CKD' dataset within the 'Nephroseq' database demonstrating **a** *NRBF2* expression in glomeruli in DKD and healthy living donor samples *n*=33 **b** *NRBF2* expression in tubular interstitium in DKD and healthy living donor samples *n*=39

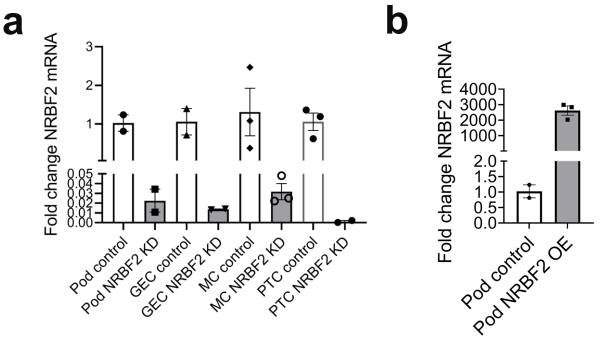

Supplementary Figure 8: Level NRBF2 knockdown and overexpression in human kidney cell lines

a qPCR data demonstrating expression of *NRBF2* mRNA in conditionally-immortalised human podocytes (Pod), Glomerular Endothelial Cells (GEC), Mesangial Cells (MC) and Proximal Tubular Cells (PTC) transduced with shRNA targetting *NRBF2* or scrambled-RNA ‘Control’.

b qPCR data confirming increased *NRBF2* mRNA in podocytes transduced with human NRBF2-containing lentivirus (i.e., ‘overexpressing’ [OE] cell system).

Selected GO Biological Process: enriched (p < 0.05, q < 0.1) in at least one cell type

* p < 0.05, ** p < 0.01

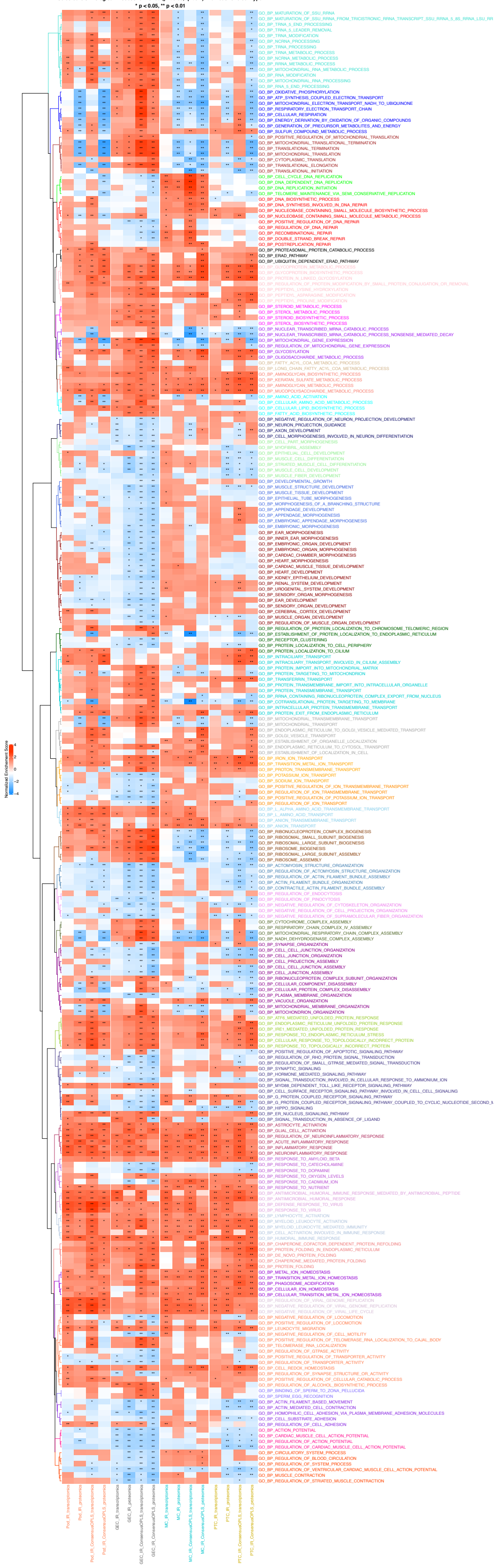

Supplementary Fig. 9: GSEA results using GOBP

Gene set enrichment analysis was performed using results of differential expression ('DE') and Consensus-Orthogonal Partial Least Squares Discriminant ('OPLS') analysis, 'insulin resistant' vs 'basal' cells. Enriched GO terms were filtered for significance in at least one cell type (nominal p-value<0.05 and q-value<0.1 from both omics data either from individual DE or Consensus OPLS). Terms were then hierarchically clustered with the semantic similarity between GO terms based on the graph structure of GO (Wang measure) using the R package GOSemSim (v2.12.1) and then displayed as a heatmap of normalised enrichment scores (NES), * p<0.05, ** p<0.01.

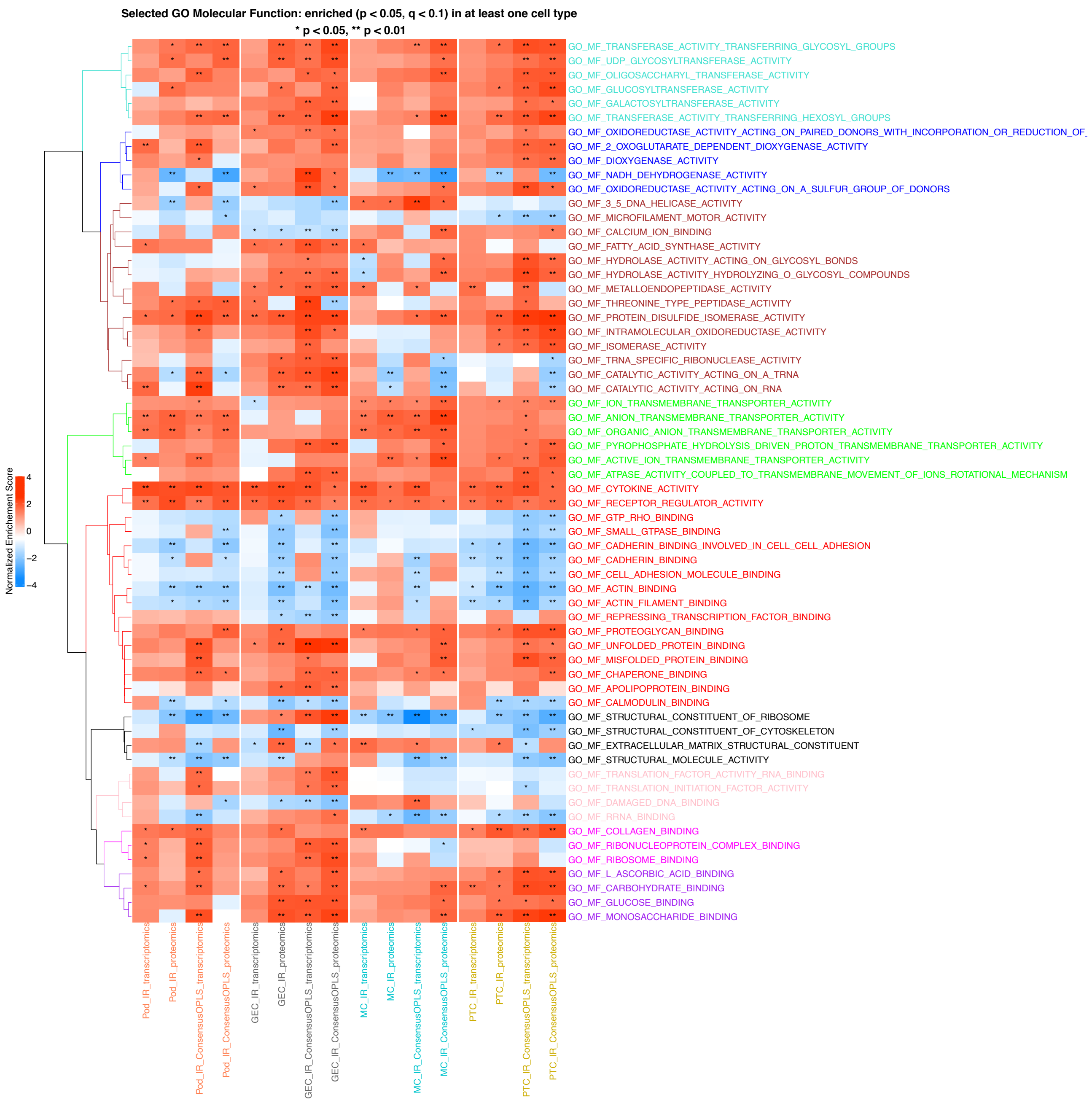

Supplementary Fig. 10: GSEA results using GOMF

Gene set enrichment analysis was performed using results of differential expression (‘DE’) and Consensus-Orthogonal Partial Least Squares Discriminant (‘OPLS’) analysis, ‘insulin resistant’ vs ‘basal’ cells. Enriched GO terms were filtered for significance in at least one cell type (nominal p-value<0.05 and q-value<0.1 from both omics data either from individual DE or Consensus OPLS). Terms were then hierarchically clustered with the semantic similarity between GO terms based on the graph structure of GO (Wang measure) using the R package GOSemSim (v2.12.1) and then displayed as a heatmap of normalised enrichment scores (NES), * p<0.05, ** p<0.01.

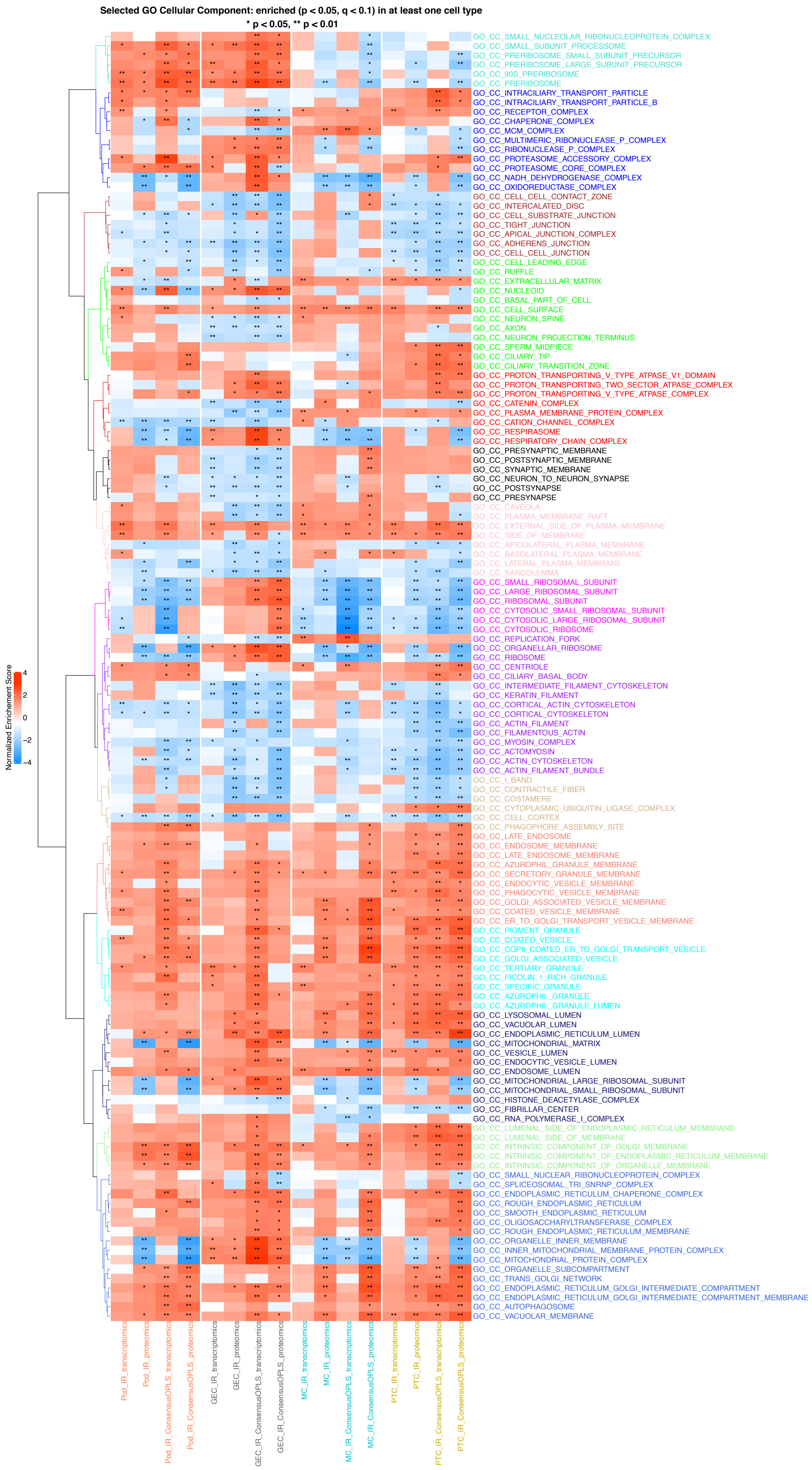
Selected GO Cellular Component: enriched (p < 0.05, q < 0.1) in at least one cell type
* p < 0.05, ** p < 0.01
Normalized Enrichement Score
4
2
0
−2
−4
GO_CC_SMALL_NUCLEOLAR_RIBONUCLEOPROTEIN_COMPLEX
GO_CC_SMALL_SUBUNIT_PROCESSOME
GO_CC_PRERIBOSOME_SMALL_SUBUNIT_PRECURSOR
GO_CC_PRERIBOSOME_LARGE_SUBUNIT_PRECURSOR
GO_CC_90S_PRERIBOSOME
GO_CC_PRERIBOSOME
GO_CC_INTRACILIARY_TRANSPORT_PARTICLE
GO_CC_INTRACILIARY_TRANSPORT_PARTICLE_B
GO_CC_RECEPTOR_COMPLEX
GO_CC_CHAPERONE_COMPLEX
GO_CC_MCM_COMPLEX
GO_CC_MULTIMERIC_RIBONUCLEASE_P_COMPLEX
GO_CC_RIBONUCLEASE_P_COMPLEX
GO_CC_PROTEASOME_ACCESSORY_COMPLEX
GO_CC_PROTEASOME_CORE_COMPLEX
GO_CC_NADH_DEHYDROGENASE_COMPLEX
GO_CC_OXIDOREDUCTASE_COMPLEX
GO_CC_CELL_CELL_CONTACT_ZONE
GO_CC_INTERCALATED_DISC
GO_CC_CELL_SUBSTRATE_JUNCTION
GO_CC_TIGHT_JUNCTION
GO_CC_APICAL_JUNCTION_COMPLEX
GO_CC_ADHERENS_JUNCTION
GO_CC_CELL_CELL_JUNCTION
GO_CC_CELL_LEADING_EDGE
GO_CC_RUFFLE
GO_CC_EXTRACELLULAR_MATRIX
GO_CC_NUCLEOID
GO_CC_BASAL_PART_OF_CELL
GO_CC_CELL_SURFACE
GO_CC_NEURON_SPINE
GO_CC_AXON
GO_CC_NEURON_PROJECTION_TERMINUS
GO_CC_SPERM_MIDPIECE
GO_CC_CILIARY_TIP
GO_CC_CILIARY_TRANSITION_ZONE
GO_CC_PROTON_TRANSPORTING_V_TYPE_ATPASE_V1_DOMAIN
GO_CC_PROTON_TRANSPORTING_TWO_SECTOR_ATPASE_COMPLEX
GO_CC_PROTON_TRANSPORTING_V_TYPE_ATPASE_COMPLEX
GO_CC_CATENIN_COMPLEX
GO_CC_PLASMA_MEMBRANE_PROTEIN_COMPLEX
GO_CC_CATION_CHANNEL_COMPLEX
GO_CC_RESPIRASOME
GO_CC_RESPIRATORY_CHAIN_COMPLEX
GO_CC_PRESYNAPTIC_MEMBRANE
GO_CC_POSTSYNAPTIC_MEMBRANE
GO_CC_SYNAPTIC_MEMBRANE
GO_CC_NEURON_TO_NEURON_SYNAPSE
GO_CC_POSTSYNAPSE
GO_CC_PRESYNAPSE
GO_CC_CAVEOLA
GO_CC_PLASMA_MEMBRANE_RAFT
GO_CC_EXTERNAL_SIDE_OF_PLASMA_MEMBRANE
GO_CC_SIDE_OF_MEMBRANE
GO_CC_APICOLATERAL_PLASMA_MEMBRANE
GO_CC_BASOLATERAL_PLASMA_MEMBRANE
GO_CC_LATERAL_PLASMA_MEMBRANE
GO_CC_SARCOLEMMA
GO_CC_SMALL_RIBOSOMAL_SUBUNIT
GO_CC_LARGE_RIBOSOMAL_SUBUNIT
GO_CC_RIBOSOMAL_SUBUNIT
GO_CC_CYTOSOLIC_SMALL_RIBOSOMAL_SUBUNIT
GO_CC_CYTOSOLIC_LARGE_RIBOSOMAL_SUBUNIT
GO_CC_CYTOSOLIC_RIBOSOME
GO_CC_REPLICATION_FORK
GO_CC_ORGANELLAR_RIBOSOME
GO_CC_RIBOSOME
GO_CC_CENTRIOLE
GO_CC_CILIARY_BASAL_BODY
GO_CC_INTERMEDIATE_FILAMENT_CYTOSKELETON
GO_CC_KERATIN_FILAMENT
GO_CC_CORTICAL_ACTIN_CYTOSKELETON
GO_CC_CORTICAL_CYTOSKELETON
GO_CC_ACTIN_FILAMENT
GO_CC_FILAMENTOUS_ACTIN
GO_CC_MYOSIN_COMPLEX
GO_CC_ACTOMYOSIN
GO_CC_ACTIN_CYTOSKELETON
GO_CC_ACTIN_FILAMENT_BUNDLE
GO_CC_I_BAND
GO_CC_CONTRACTILE_FIBER
GO_CC_COSTAMERE
GO_CC_CYTOPLASMIC_UBIQUITIN_LIGASE_COMPLEX
GO_CC_CELL_CORTEX
GO_CC_PHAGOPHORE_ASSEMBLY_SITE
GO_CC_LATE_ENDOSOME
GO_CC_ENDOSOME_MEMBRANE
GO_CC_LATE_ENDOSOME_MEMBRANE
GO_CC_AZUROPHIL_GRANULE_MEMBRANE
GO_CC_SECRETORY_GRANULE_MEMBRANE
GO_CC_ENDOCYTIC_VESICLE_MEMBRANE
GO_CC_PHAGOCYTIC_VESICLE_MEMBRANE
GO_CC_GOLGI_ASSOCIATED_VESICLE_MEMBRANE
GO_CC_COATED_VESICLE_MEMBRANE
GO_CC_ER_TO_GOLGI_TRANSPORT_VESICLE_MEMBRANE
GO_CC_PIGMENT_GRANULE
GO_CC_COATED_VESICLE
GO_CC_COPII_COATED_ER_TO_GOLGI_TRANSPORT_VESICLE
GO_CC_GOLGI_ASSOCIATED_VESICLE
GO_CC_TERTIARY_GRANULE
GO_CC_FICOLIN_1_RICH_GRANULE
GO_CC_SPECIFIC_GRANULE
GO_CC_AZUROPHIL_GRANULE
GO_CC_AZUROPHIL_GRANULE_LUMEN
GO_CC_LYSOSOMAL_LUMEN
GO_CC_VACUOLAR_LUMEN
GO_CC_ENDOPLASMIC_RETICULUM_LUMEN
GO_CC_MITOCHONDRIAL_MATRIX
GO_CC_VESICLE_LUMEN
GO_CC_ENDOCYTIC_VESICLE_LUMEN
GO_CC_ENDOSOME_LUMEN
GO_CC_MITOCHONDRIAL_LARGE_RIBOSOMAL_SUBUNIT
GO_CC_MITOCHONDRIAL_SMALL_RIBOSOMAL_SUBUNIT
GO_CC_HISTONE_DEACETYLASE_COMPLEX
GO_CC_FIBRILLAR_CENTER
GO_CC_RNA_POLYMERASE_I_COMPLEX
GO_CC_LUMENAL_SIDE_OF_ENDOPLASMIC_RETICULUM_MEMBRANE
GO_CC_LUMENAL_SIDE_OF_MEMBRANE
GO_CC_INTRINSIC_COMPONENT_OF_GOLGI_MEMBRANE
GO_CC_INTRINSIC_COMPONENT_OF_ENDOPLASMIC_RETICULUM_MEMBRANE
GO_CC_INTRINSIC_COMPONENT_OF_ORGANELLE_MEMBRANE
GO_CC_SMALL_NUCLEAR_RIBONUCLEOPROTEIN_COMPLEX
GO_CC_SPLICEOSOMAL_TRI_SNRNP_COMPLEX
GO_CC_ENDOPLASMIC_RETICULUM_CHAPERONE_COMPLEX
GO_CC_ROUGH_ENDOPLASMIC_RETICULUM
GO_CC_SMOOTH_ENDOPLASMIC_RETICULUM
GO_CC_OLIGOSACCHARYLTRANSFERASE_COMPLEX
GO_CC_ROUGH_ENDOPLASMIC_RETICULUM_MEMBRANE
GO_CC_ORGANELLE_INNER_MEMBRANE
GO_CC_INNER_MITOCHONDRIAL_MEMBRANE_PROTEIN_COMPLEX
GO_CC_MITOCHONDRIAL_PROTEIN_COMPLEX
GO_CC_ORGANELLE_SUBCOMPARTMENT
GO_CC_TRANS_GOLGI_NETWORK
GO_CC_ENDOPLASMIC_RETICULUM_GOLGI_INTERMEDIATE_COMPARTMENT
GO_CC_ENDOPLASMIC_RETICULUM_GOLGI_INTERMEDIATE_COMPARTMENT_MEMBRANE
GO_CC_AUTOPHAGOSOME
GO_CC_VACUOLAR_MEMBRANE
Pod_IR_transcriptomics
Pod_IR_proteomics
Pod_IR_ConsensusOPLS_transcriptomics
Pod_IR_ConsensusOPLS_proteomics
GEC_IR_transcriptomics
GEC_IR_proteomics
GEC_IR_ConsensusOPLS_transcriptomics
GEC_IR_ConsensusOPLS_proteomics
MC_IR_transcriptomics
MC_IR_proteomics
MC_IR_ConsensusOPLS_transcriptomics
MC_IR_ConsensusOPLS_proteomics
PTC_IR_transcriptomics
PTC_IR_proteomics
PTC_IR_ConsensusOPLS_transcriptomics
PTC_IR_ConsensusOPLS_proteomics

Supplementary Fig. 11: GSEA results using GOCC

Gene set enrichment analysis was performed using results of differential expression ('DE') and Consensus-Orthogonal Partial Least Squares Discriminant ('OPLS') analysis, 'insulin resistant' vs 'basal' cells. Enriched GO terms were filtered for significance in at least one cell type (nominal p-value<0.05 and q-value<0.1 from both omics data either from individual DE or Consensus OPLS). Terms were then hierarchically clustered with the semantic similarity between GO terms based on the graph structure of GO (Wang measure) using the R package GOSemSim (v2.12.1) and then displayed as a heatmap of normalised enrichment scores (NES), * p<0.05, ** p<0.01.

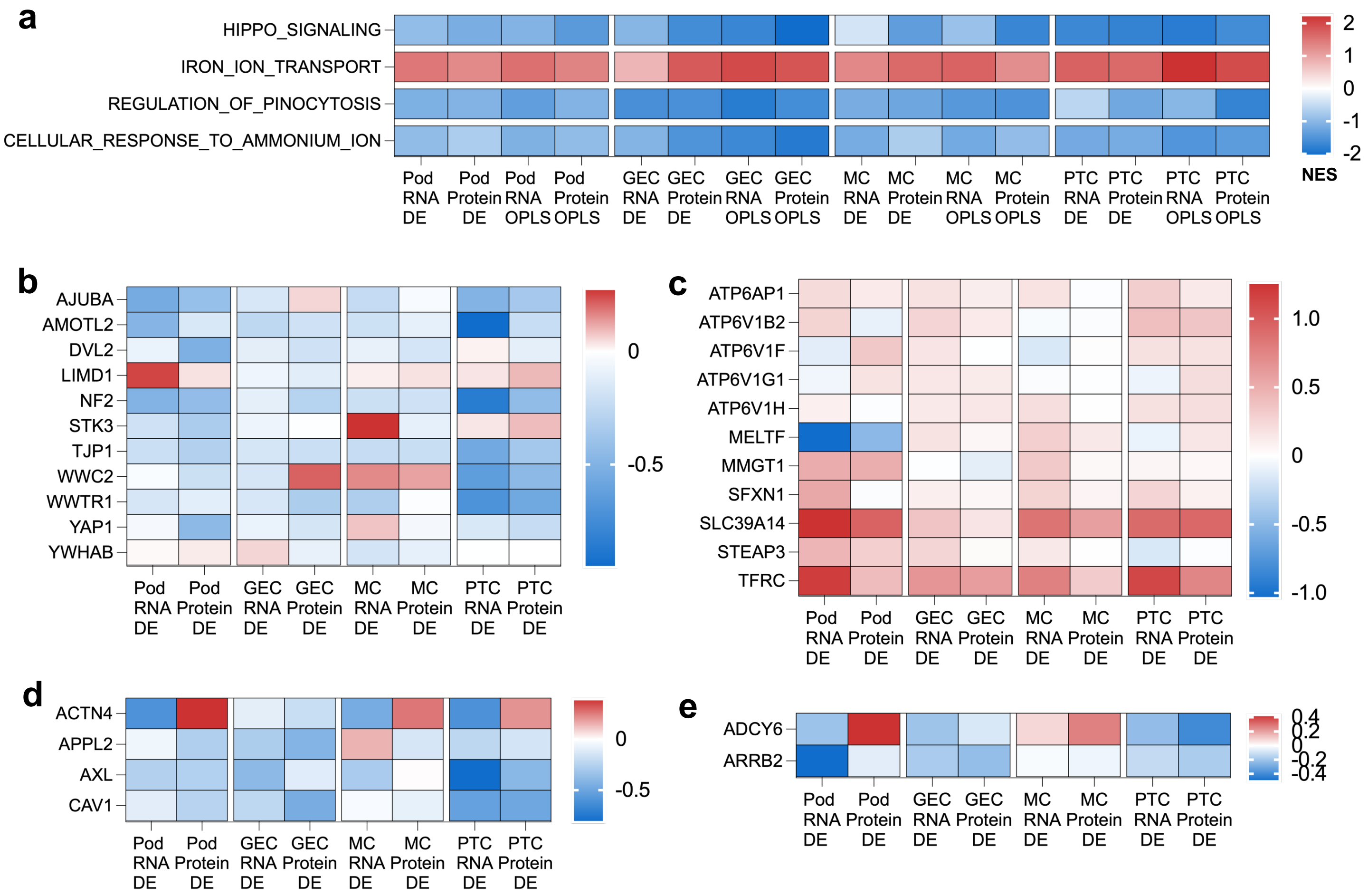

Supplementary Fig. 12: Consistently regulated GOBP terms in insulin resistant kidney cells

Gene set enrichment analysis was performed using results of differential expression ('DE') and Consensus-Orthogonal Partial Least Squares Discriminant ('OPLS') analysis. Heatmaps displaying **a** normalised enrichment scores ('NES') for GOBP terms commonly regulated in insulin resistant podocytes (Pods), Glomerular Endothelial Cells (GECs), Mesangial Cells (MCs) and Proximal Tubular Cells (PTCs) at both the RNA and protein level and **b-e** CORE enrichment genes for **b** 'HIPPO signalling' **c** 'Iron Ion Transport' **d** 'Regulation of pinocytosis' and **e** 'Cellular response to ammonium ion'

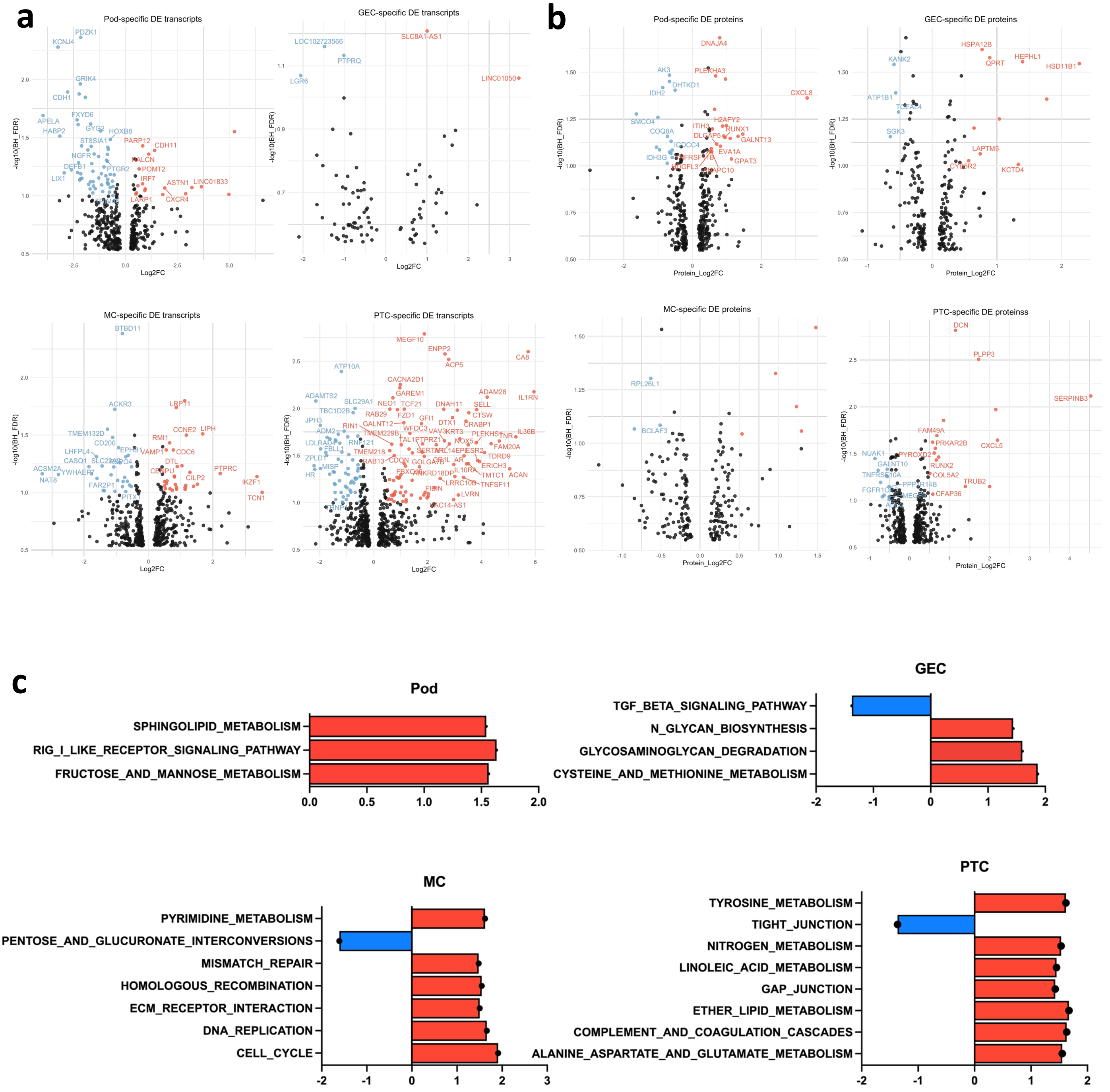

Supplementary Fig. 13: Individual analysis to reveal transcripts and proteins regulated in cell-line specific patterns

Transcriptome and proteome data were individually normalised and analysed for each cell line. Cell-line-specific regulation of transcripts and proteins was established using nominal $p<0.05$ and further filtering such that, in addition to $p>0.05$ in the other cell lines, Log2FC <0.1 for upregulated and >-0.1 for downregulated transcripts/ proteins. Volcano plots display the **a** transcripts and **b** proteins that were up- or down-regulated in a cell-line specific manner; -log10 FDR (Benjamini Hochberg) vs Log2 fold-change. **c** Gene Set Enrichment Analysis using the KEGG database demonstrating the cell-type-specific enriched pathways in response to insulin resistance in podocytes (Pods), Glomerular Endothelial Cells (GECs), Mesangial Cells (MCs) and Proximal Tubular Cells (PTCs) (p<0.05).

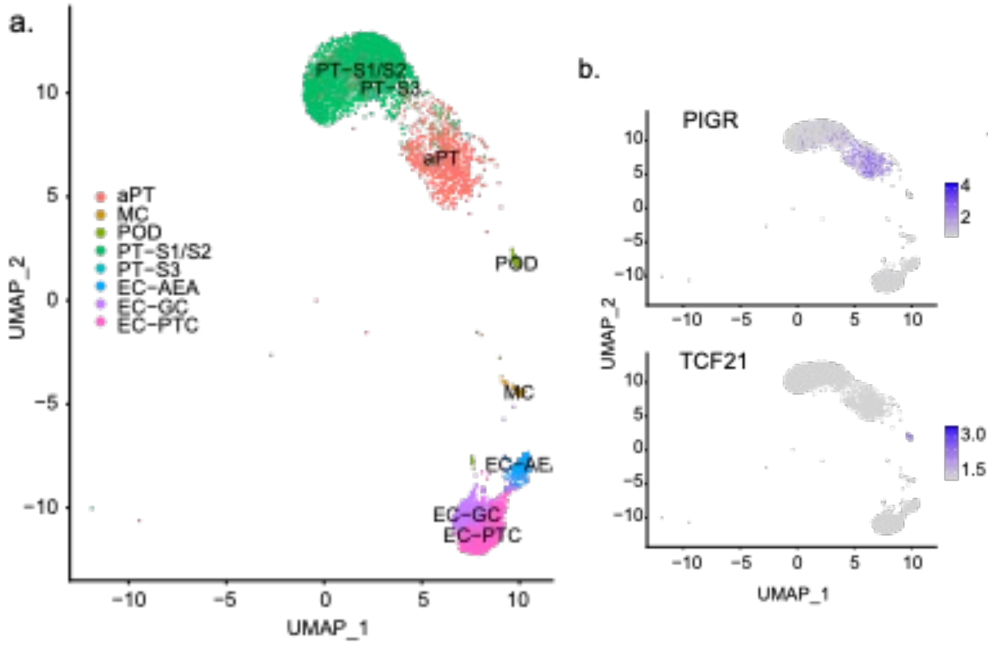

Supplementary Fig. 14: Targeted analysis of human kidney single-cell sequencing demonstrates cell-type-specific expression of genes of interest.
a Reference UMAP of healthy human kidney single-cell sequencing data from KPMP highlighting specific cell type clusters of interest (‘aPT’ = Proximal Tubule Cell-adaptive; ‘MC’ = Mesangial cells; ‘POD’ = Podocytes; ‘PT-S1/S2’ = Proximal Tubule Subcluster 1 /2; ‘PT-S3’ = Proximal Tubule Subcluster 3; ‘EC-AEA’ = Endothelial Cell-Arteriola; ‘EC-GC’ = Endothelial Cell-Glomerular Capillary; ‘EC-PTC’ = Endothelial Cell-Peritubular Capillary), **b** UMAP plots displaying cell specificity of expression (in healthy human kidney) for selected genes found to be regulated at cell-line-specific level in response to insulin resistance.

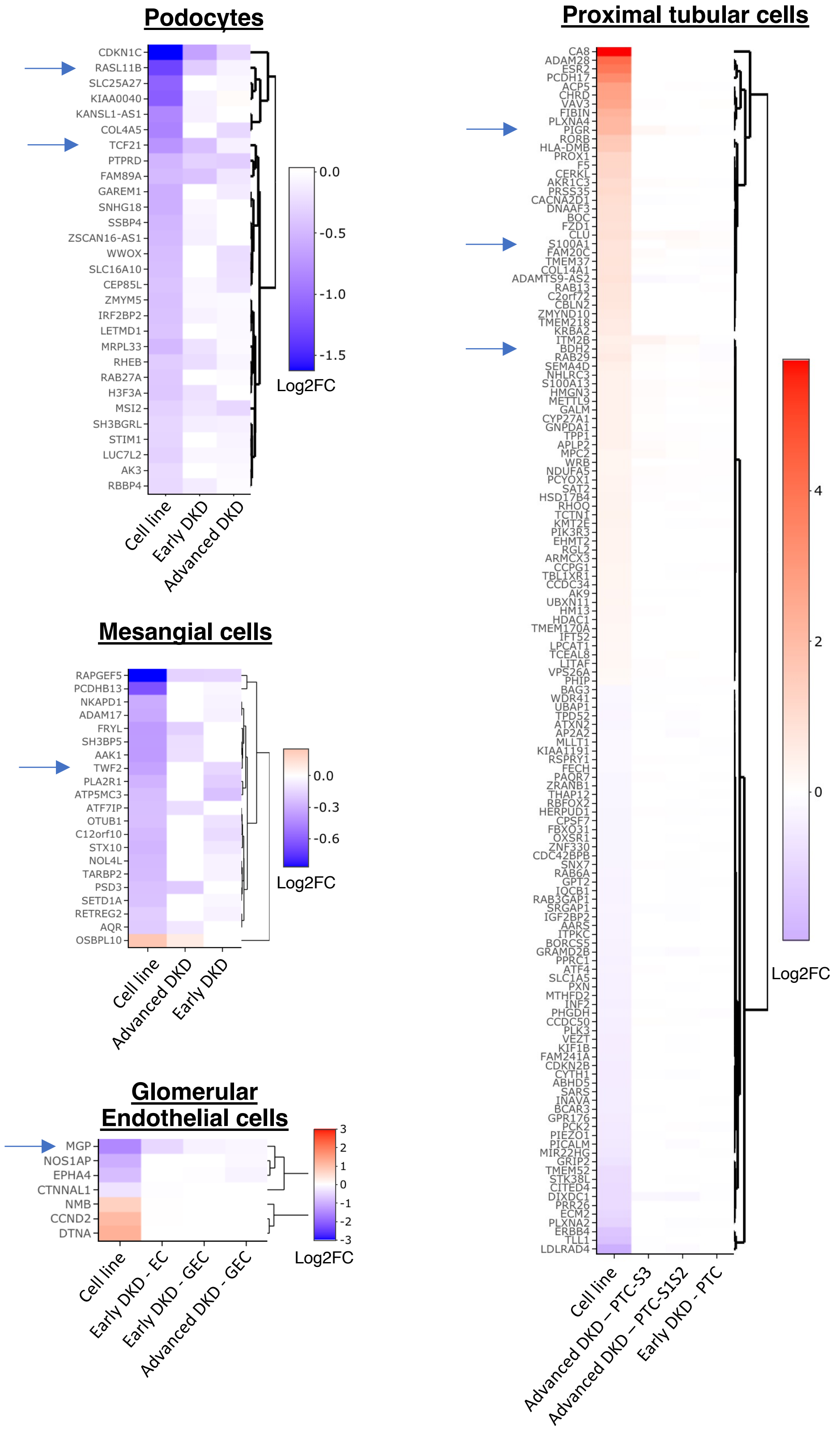

Supplementary Fig. 15: Overlap between cell-type-specific responses to insulin resistance (*in vitro*) and human diabetic kidney disease

Log_2 Fold Change values for genes found to be significantly regulated in insulin resistance, in a cell-type-specific manner *in vitro* that are also significantly regulated in equivalent cell types in human diabetic kidney disease, single-cell-RNA sequencing data (adjusted $p < 0.05$). 'Early DKD' = an American Indian type-2 diabetes cohort with early-DKD (n=44 early-DKD vs n=18 LD); 'Advanced DKD' = data from the Kidney Precision Medicine Project (n=10 advanced-DKD vs n=18 living donor); 'EC' = Endothelial Cell; 'GEC' = Glomerular Endothelial Cell subcluster; 'PTC-S3' = Proximal Tubule Subcluster 3'; 'PTC-S1S2' = Proximal Tubule Subcluster 1 / 2'

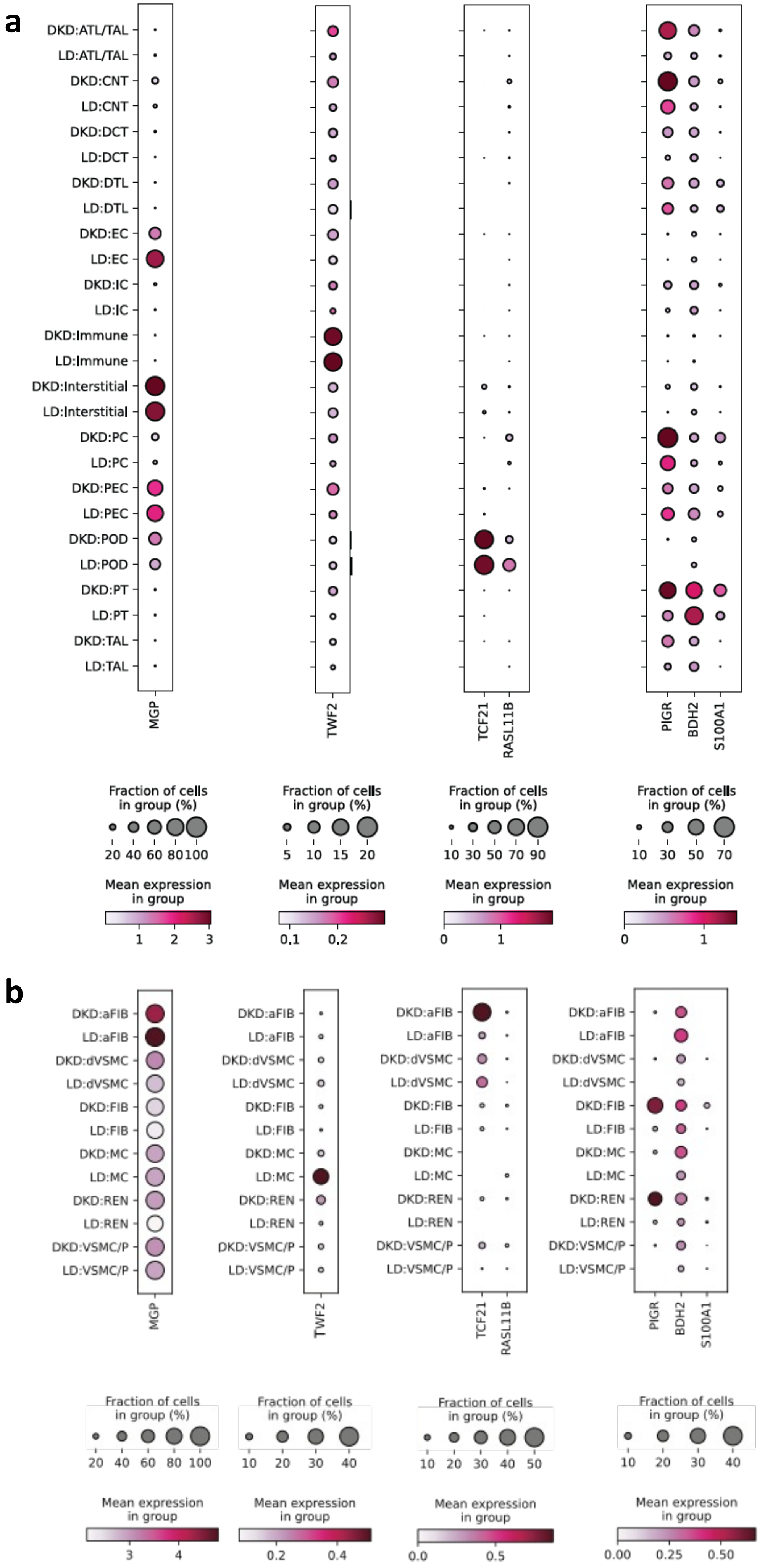
a
DKD:ATL/TAL
LD:ATL/TAL
DKD:CNT
LD:CNT
DKD:DCT
LD:DCT
DKD:DTL
LD:DTL
DKD:EC
LD:EC
DKD:IC
LD:IC
DKD:Immune
LD:Immune
DKD:Interstitial
LD:Interstitial
DKD:PC
LD:PC
DKD:PEC
LD:PEC
DKD:POD
LD:POD
DKD:PT
LD:PT
DKD:TAL
LD:TAL
MGP
TWF2
TCF21
RASL11B
PIGR
BDH2
S100A1
Fraction of cells in group (%)
20 40 60 80 100
5 10 15 20
10 30 50 70 90
10 30 50 70
Mean expression in group
1 2 3
0.1 0.2
0 1
0 1
b
DKD:aFIB
LD:aFIB
DKD:dVSMC
LD:dVSMC
DKD:FIB
LD:FIB
DKD:MC
LD:MC
DKD:REN
LD:REN
DKD:VSMC/P
LD:VSMC/P
MGP
TWF2
TCF21
RASL11B
PIGR
BDH2
S100A1
Fraction of cells in group (%)
20 40 60 80 100
10 20 30 40
10 20 30 40 50
10 20 30 40
Mean expression in group
3 4
0.2 0.4
0.0 0.5
0.00 0.25 0.50

Supplementary Fig. 16: Targeted analysis of kidney single cell sequencing data highlights replicated cell-type-specific responses to insulin resistance in human diabetic kidney disease

a Single-cell sequencing analysis of target genes in each cell-type cluster (dot plots displaying the percentage of expressing cells and mean expression values) in advanced DKD from the Kidney Precision Medicine Project (*n*=10 advanced-DKD vs *n*=18 living donor) and **b** expression of those genes in ‘interstitial cell’ subclusters.

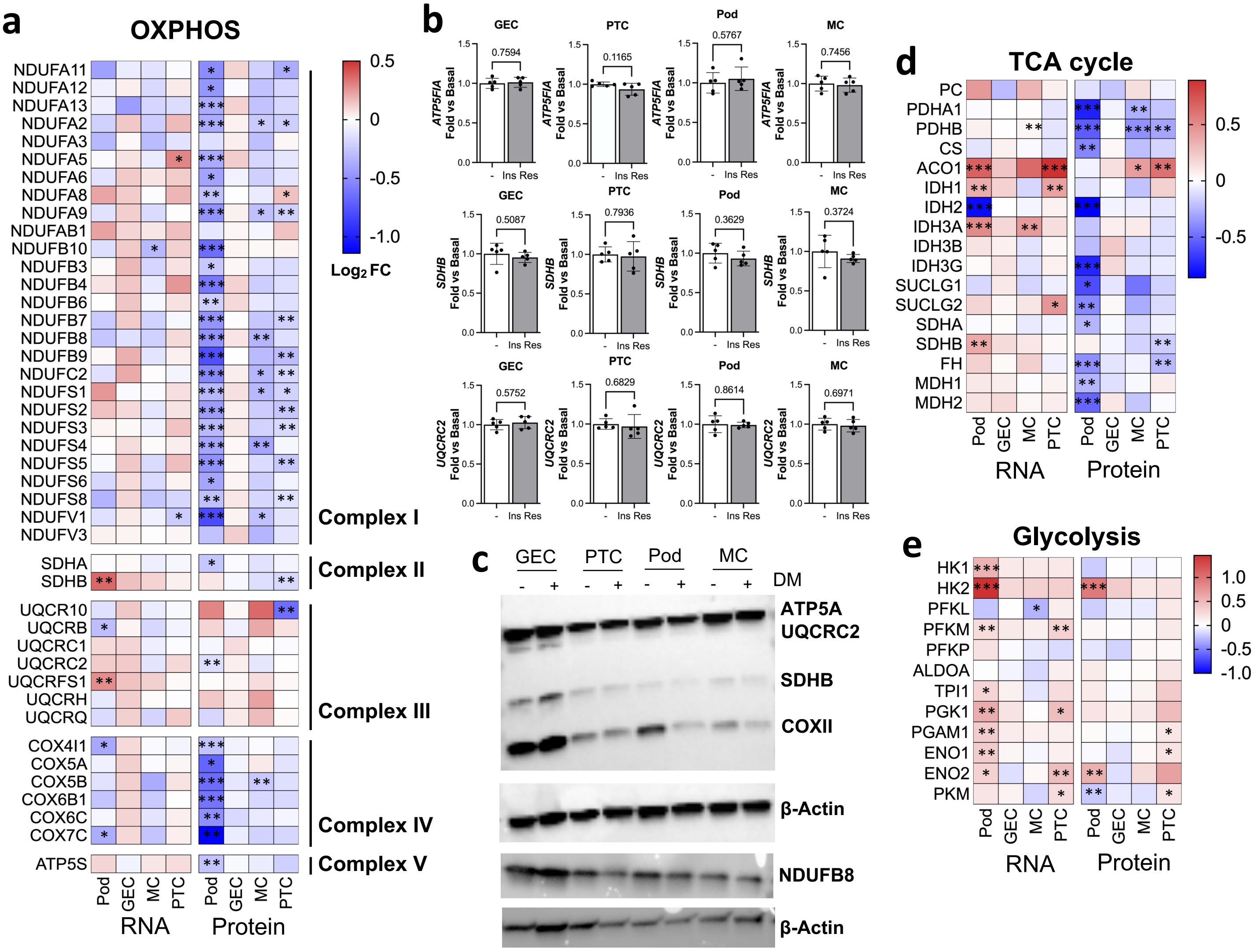

Supplementary Fig. 17: Differential expression of OXPHOS, TCA cycle and glycolysis transcripts and proteins in insulin resistant vs insulin sensitive cell lines

Log_2 Fold Change values in insulin resistant vs control conditions for **a** respiratory chain (OXPHOS) complex transcripts and proteins detected in all cell types *p<0.05, **p<0.01, ***p<0.001, unadjusted **b** qPCR results to show expression of mitochondrial OXPHOS subunits at the mRNA level (n=5) and **c** Western blotting demonstrating expression of equivalent OXPHOS protein subunits in 'basal' vs 'insulin resistant' conditionally immortalised human glomerular endothelial cells (GECs), proximal tubular cells (PTCs), podocytes (Pods) and mesangial cells (MCs) (n=5), two-tailed *t*-test **d** TCA cycle components detected at both the transcript and protein level in all cell types, **e** glycolysis enzymes detected at both the transcript and protein level in all cell types *p<0.05, **p<0.01, ***p<0.001, unadjusted

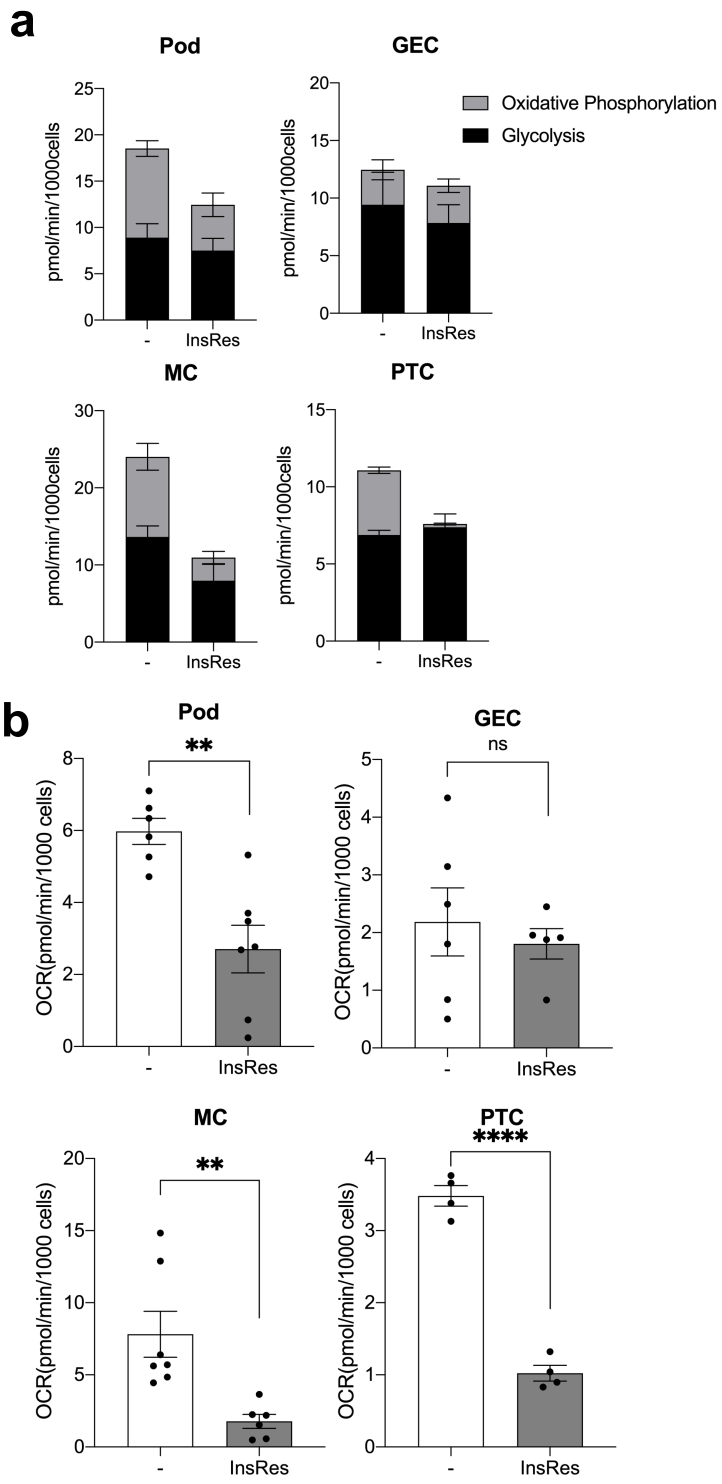

Supplementary Fig. 18: Mitochondrial metabolism is differentially impaired in insulin-resistant kidney cells

a ATP production attributed to glycolysis or oxidative phosphorylation in each cell line under basal or insulin resistant conditions, **b** OCR values per minute, per 1000 cells representing maximal respiration in 'Insulin Resistant' vs 'Basal' conditions in Podocytes ('Pod', *n*=6), Glomerular Endothelial Cells ('GEC', *n*=5 or 6), Mesangial Cells ('MC', *n*=6) and Proximal Tubular Cells (PTC, *n*=4) ***p*<0.01, ****p*<0.001, *****p*<0.0001, two-tailed *t*-test.

Uncropped Western blots for Supplementary material

Uncropped gels for Supplementary Figure 1

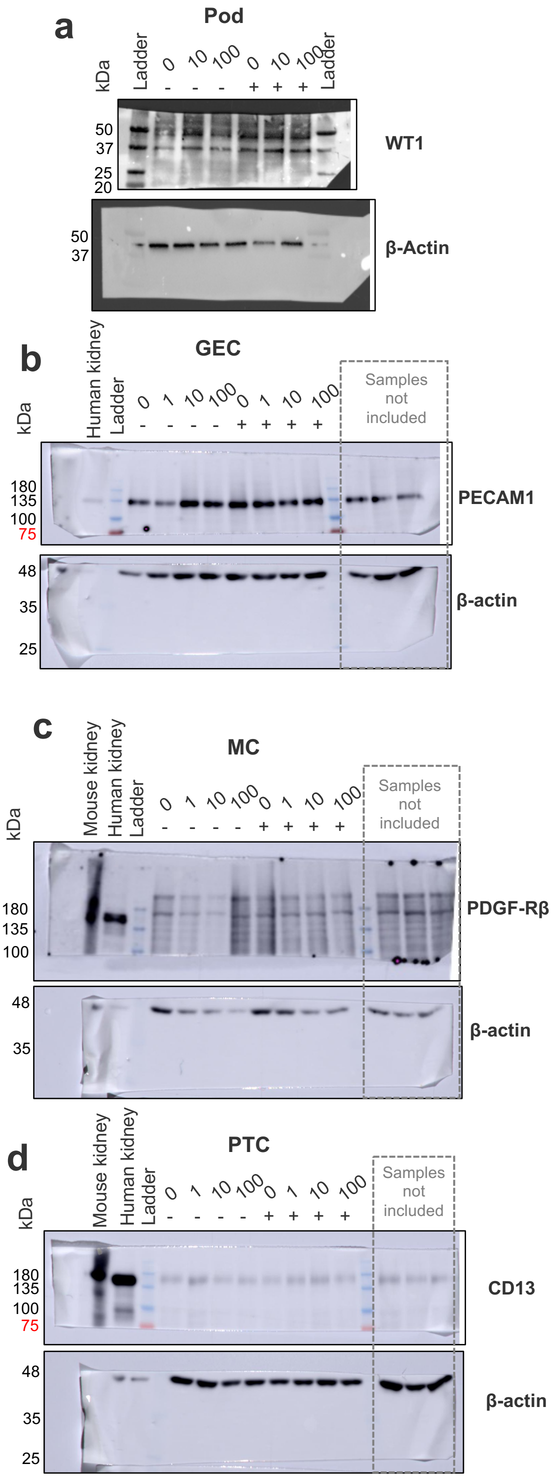

Uncropped gels for Supplementary Figure 17

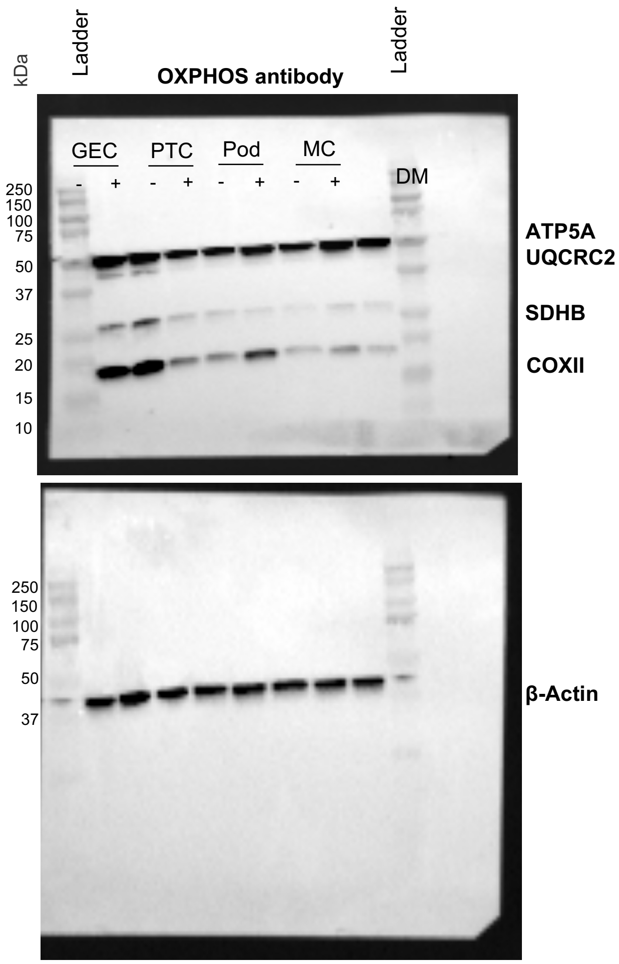

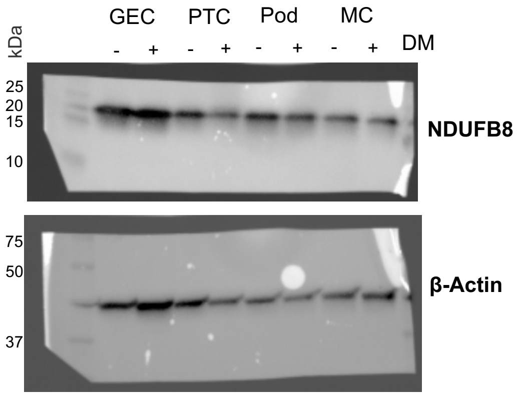

Supplement: Supplementary file 1 — Supplementary Information [file 41467_2024_54089_MOESM1_ESM.pdf]
