# Supplementary material for: Profiling of insulin-resistant kidney models and human biopsies reveals common and cell-type-specific mechanisms underpinning Diabetic Kidney Disease
Source: Nat Commun. 2024 Nov 19;15:10018. doi: 10.1038/s41467-024-54089-1 (PMC11576882; doi:10.1038/s41467-024-54089-1)
Supplement: Supplementary file 2 — Description of Additional Supplementary Files [file 41467_2024_54089_MOESM2_ESM.pdf]

Description of Additional Supplementary Files

Supplementary Data 1: Top 40 consistent genes and their functions

Combined results of DE (Log2 FC) and consensus-OPLS predicted values were used to select the 'Top 40' consistently regulated proteins and transcripts across all cell lines. Functional annotations were downloaded from the Uniprot and/or EBI database and edited manually.

Supplementary Data 2: Transcription Factor binding site enrichment for the top 40 consistently regulated genes

Results of Overrepresentation analysis (one tailed) using the TRANSFAC database demonstrating transcription factor binding sites significantly enriched amongst top 40 genes, consistently regulated across all insulin resistant cell types.

Supplementary Data 3: Baseline characteristics of human cohorts

Baseline characteristics of human Diabetic Kidney Disease cohorts, age, diabetes duration, HbA1C, GFR, ACR and Proteinuria presented as mean (SD)

Supplementary Data 4: Immune GSEA results

Normalised enrichment scores (NES) for immune/ inflammatory response pathways significantly enriched across insulin resistant cell lines and the core enrichment inflammatory genes and proteins.

Supplementary Data 5: ER stress GSEA results

Normalised enrichment scores (NES) for ER stress/UPR pathways significantly enriched across insulin resistant cell lines and the core enrichment ER stress/UPR genes and proteins.

Supplementary Data 6: Glycoprotein metabolism GSEA results

Normalised enrichment scores (NES) for pathways related to glycoprotein metabolism and the core enrichment genes and proteins, that were found to be significantly enriched across insulin resistant cell lines.

Supplementary Data 7: CORE mitochondrial genes driving enrichment

Core genes and proteins driving the enrichment of mitochondrial pathways were selected from GSEA results. Functional annotations were downloaded from the Uniprot database and edited manually.

Supplementary Data 8: KPMP IDs

Participant ID numbers for Diabetic Kidney Disease patients from the Kidney Precision medicine project that were used in this study.
