# Supplementary material for: Profiling of insulin-resistant kidney models and human biopsies reveals common and cell-type-specific mechanisms underpinning Diabetic Kidney Disease
Source: Nat Commun. 2024 Nov 19;15:10018. doi: 10.1038/s41467-024-54089-1 (PMC11576882; doi:10.1038/s41467-024-54089-1)
Supplement: Supplementary file 11 — Reporting Summary [file 41467_2024_54089_MOESM11_ESM.pdf]

nature portfolio

Corresponding author(s): Professor Richard Coward

Last updated by author(s): Sep 24, 2024

Reporting Summary

Nature Portfolio wishes to improve the reproducibility of the work that we publish. This form provides structure for consistency and transparency in reporting. For further information on Nature Portfolio policies, see our Editorial Policies and the Editorial Policy Checklist.

Statistics

For all statistical analyses, confirm that the following items are present in the figure legend, table legend, main text, or Methods section.

n/a	Confirmed	
☐	☒	The exact sample size (*n*) for each experimental group/condition, given as a discrete number and unit of measurement
☐	☒	A statement on whether measurements were taken from distinct samples or whether the same sample was measured repeatedly
☐	☒	The statistical test(s) used AND whether they are one- or two-sided<br>*Only common tests should be described solely by name; describe more complex techniques in the Methods section.*
☒	☐	A description of all covariates tested
☐	☒	A description of any assumptions or corrections, such as tests of normality and adjustment for multiple comparisons
☐	☒	A full description of the statistical parameters including central tendency (e.g. means) or other basic estimates (e.g. regression coefficient) AND variation (e.g. standard deviation) or associated estimates of uncertainty (e.g. confidence intervals)
☐	☒	For null hypothesis testing, the test statistic (e.g. *F*, *t*, *r*) with confidence intervals, effect sizes, degrees of freedom and *P* value noted<br>*Give P values as exact values whenever suitable.*
☒	☐	For Bayesian analysis, information on the choice of priors and Markov chain Monte Carlo settings
☐	☒	For hierarchical and complex designs, identification of the appropriate level for tests and full reporting of outcomes
☐	☒	Estimates of effect sizes (e.g. Cohen's *d*, Pearson's *r*), indicating how they were calculated

Our web collection on statistics for biologists contains articles on many of the points above.

Software and code

Policy information about availability of computer code

Data collection	Details are provided throughout methods and codes can be found at https://github.com/sib-swiss/BEAt_DKD. See additional data availability statement.
Data analysis	As above. Code can be found at https://github.com/sib-swiss/BEAt_DKD.

For manuscripts utilizing custom algorithms or software that are central to the research but not yet described in published literature, software must be made available to editors and reviewers. We strongly encourage code deposition in a community repository (e.g. GitHub). See the Nature Portfolio guidelines for submitting code & software for further information.

Data

Policy information about availability of data

All manuscripts must include a data availability statement. This statement should provide the following information, where applicable:

- Accession codes, unique identifiers, or web links for publicly available datasets
- A description of any restrictions on data availability
- For clinical datasets or third party data, please ensure that the statement adheres to our policy

Data availability
The codes are available at https://github.com/sib-swiss/BEAt_DKD. The transcriptomic and proteomic datasets from insulin-sensitive and insulin-resistant cell lines are submitted and will be made publicly available to NCBI under the BioProject PRJNA905899. Additional data used in this manuscript are accessible at https://

epdc.sib.swiss (European Platform for Diabetes and Complications) and https://atlas.kpmp.org/repository (Kidney Precision Medicine Project). All participants from human cohort provided informed consent. Due to privacy protection concerns, individual-level genotype, and gene expression data from the early DKD study cannot be made publicly available.
Other data can be found in the 'Source data file' accompanying this manuscript.

Research involving human participants, their data, or biological material

Policy information about studies with human participants or human data. See also policy information about sex, gender (identity/presentation), and sexual orientation and race, ethnicity and racism.

Reporting on sex and gender	Our studies involve both male and female participants, demographic details can be found in Supplementary table 3. For our analysis of human cohorts, individual-level data cannot be made available.
Reporting on race, ethnicity, or other socially relevant groupings	We used data from data from the European Renal cDNA Bank-Kroener-Fresenius biopsy bank), an American Indian type 2 diabetes cohort, and Kidney Precision Medicine Project.<br>No information on ancestry is available
Population characteristics	Demographic details of participants are provided in supplementary table 3.
Recruitment	Samples from the American Indian type 2 diabetes cohort consist of protocol human kidney biopsies from individuals with type 2 diabetes from the Gila River American Indian Community. The study participants were enrolled in a randomised, double-blinded, placebo-controlled interventional clinical trial funded by the National Institute of Diabetes and Digestive and Kidney Diseases (NIDDK). Biopsy samples in ERCB were obtained from patients after informed consent and stratified by the reference pathologist of the ERCB according to their histological diagnoses.
Ethics oversight	The studies were conducted in accordance with the tenets of the Declaration of Helsinki and approved by Institutional Review Board of the National Institute of Diabetes and Digestive and Kidney Diseases (NIDDK). Written informed consent was obtained from all participants.

Note that full information on the approval of the study protocol must also be provided in the manuscript.

Field-specific reporting

Please select the one below that is the best fit for your research. If you are not sure, read the appropriate sections before making your selection.

[x] Life sciences [ ] Behavioural & social sciences [ ] Ecological, evolutionary & environmental sciences

For a reference copy of the document with all sections, see nature.com/documents/nr-reporting-summary-flat.pdf

Life sciences study design

All studies must disclose on these points even when the disclosure is negative.

Sample size	All sample sizes are given in the respective figure legends and/or methods. For in vitro work, no sample size calculations were performed, sample sizes were chosen based on previous experience with in vitro cell experiments and based on standard practice. For our analysis using human transcriptomic data, we used the maximum sample size available to us in each cohort and as such, no power calculations were performed.
Data exclusions	No data were excluded from our analyses.
Replication	Our primary datasets and analyses were performed on in vitro models. Any significant findings were validated in human datasets. We focused our results and follow-up experimental studies on gene expression changes that were consistent between in vitro data and human cohorts.
Randomization	Samples were randomized to batches for RNAseq and proteomics (mass spectrometry) runs. For functional follow-up experiments, randomization was not fully possible as samples were allocated into experimental groups based on culture condition (i.e., 'basal' or 'diabetic'), although samples were harvested and analysed in a randomised manner where possible. In functional experiments (cell lines) there are no covariates to control for.
Blinding	RNA sequencing and mass spectrometry were performed by individuals blinded to experimental group. Blinding was not possible for functional follow-up experiments.

Reporting for specific materials, systems and methods

We require information from authors about some types of materials, experimental systems and methods used in many studies. Here, indicate whether each material, system or method listed is relevant to your study. If you are not sure if a list item applies to your research, read the appropriate section before selecting a response.

Materials & experimental systems

n/a	Involved in the study	
☐	☒	Antibodies
☐	☒	Eukaryotic cell lines
☒	☐	Palaeontology and archaeology
☒	☐	Animals and other organisms
☐	☒	Clinical data
☒	☐	Dual use research of concern
☒	☐	Plants

Methods

n/a	Involved in the study	
☒	☐	ChIP-seq
☒	☐	Flow cytometry
☒	☐	MRI-based neuroimaging

Antibodies

Antibodies used

Total Akt (Cell Signalling technologies #2920)
phospho-Akt (S473) (Cell Signalling technologies #4060)
Phospho-IGF-I Receptor β (Tyr1135/1136) / Insulin Receptor β (Tyr1150/1151) (Cell Signalling technologies #3024)
Total Insulin Receptor β (Cell Signalling technologies #3025)
NDUFB8 (Cell Signalling technologies #73951).
ATP5A, UQCRC2, SDHB and COXII were targeted using a total human OXPHOS antibody cocktail (Abcam ab110411).

Validation

Cell Signalling technologies provide extensive validation of their products.
Details from Cell Signalling technologies (https://www.cellsignal.com/about-us/cst-antibody-validation-principles) are as follows "Antibody signal is measured in model systems with known presence/absence of target signal. Includes wild-type vs. genetic knockout, targeted induction or silencing. Antibody signal strength is measured in cell lines or tissues representing a known continuum of target expression levels. Includes siRNA and heterozygous knockout assays. Antibody signal is correlated to target expression in model systems measured using antibody independent assays. Includes mass spectrometry and in situ hybridization. Antibody signal is compared to the signal observed using antibodies targeting nonoverlapping epitopes of the target. Includes IP, ChIP, and ChIP-seq. Antibody signal is evaluated in cell lines following heterologous expression of native (or mutated) target protein. Antibody specificity may be validated using complementary assays. Includes competitive ELISA, peptide dot blots, peptide blocking, or protein arrays."
Validation, and characterization processes are also confirmed by abcam

Eukaryotic cell lines

Policy information about cell lines and Sex and Gender in Research

Cell line source(s)	Conditionally- immortalied cell lines were derived from human kidney.
Authentication	Cell lines were authenticated using western blotting
Mycoplasma contamination	All cell lines tested negative for mycoplasma contamination at time of study.
Commonly misidentified lines (See ICLAC register)	N/A

Clinical data

Policy information about clinical studies

All manuscripts should comply with the ICMJEguidelines for publication of clinical research and a completedCONSORT checklist must be included with all submissions.

Clinical trial registration	This is not a clinical trial.
Study protocol	N/a
Data collection	Samples from the American Indian type 2 diabetes cohort consist of protocol human kidney biopsies from individuals with type 2 diabetes from the Gila River American Indian Community. The study participants were enrolled in a randomised, double-blinded, placebo-controlled interventional clinical trial funded by the National Institute of Diabetes and Digestive and Kidney Diseases (NIDDK)
Outcomes	N/A

Plants

Seed stocks	N/A
Novel plant genotypes	N/A
Authentication	N/A
